# Bis{2-bromo-4-chloro-6-[2-(phenyl­sulfon­yl)hydrazonometh­yl]phenolato-κ^2^ *N*,*O* ^1^}copper(II)

**DOI:** 10.1107/S1600536808022022

**Published:** 2008-07-19

**Authors:** Juahir Yusnita, Hapipah M. Ali, Subramaniam Puvaneswary, Ward T. Robinson, Seik Weng Ng

**Affiliations:** a Department of Chemistry, University of Malaya, 50603 Kuala Lumpur, Malaysia

## Abstract

The Cu atom in the title compound, [Cu(C_13_H_9_BrClN_2_O_3_S)_2_], is chelated by two deprotonated Schiff base ligands in a square-planar coordination geometry; the Cu atom lies on a center of inversion. The –NH– group of one anion forms an intra­molecular hydrogen bond to the phenolate atom of the symmetry-related ion.

## Related literature

For the structure of the copper derivative of a similar Schiff base ligand, see: Ali *et al.* (2007 ▶).
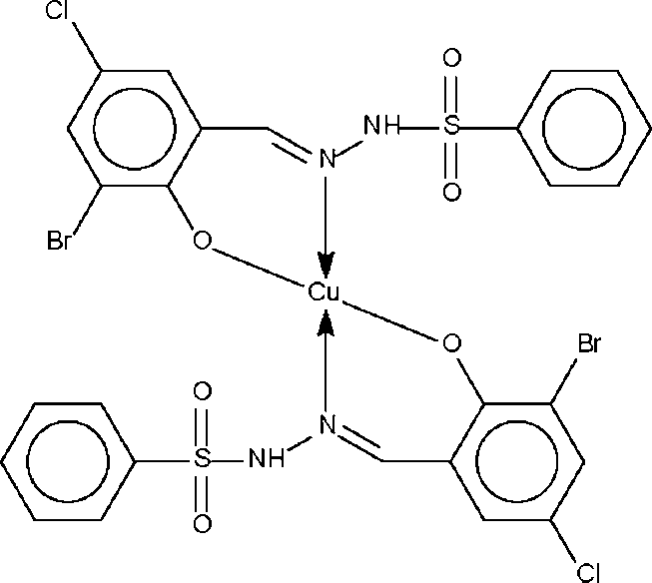


## Experimental

### 

#### Crystal data

[Cu(C_13_H_9_BrClN_2_O_3_S)_2_]
*M* *_r_* = 840.82
Triclinic, 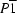

*a* = 8.0688 (1) Å
*b* = 8.2755 (1) Å
*c* = 11.7386 (2) Å
α = 95.955 (1)°
β = 90.133 (1)°
γ = 115.159 (1)°
*V* = 704.70 (2) Å^3^
*Z* = 1
Mo *K*α radiation
μ = 4.00 mm^−1^
*T* = 100 (2) K
0.20 × 0.09 × 0.09 mm

#### Data collection

Bruker SMART APEX diffractometer
Absorption correction: multi-scan (*SADABS*; Sheldrick, 1996 ▶) *T*_min_ = 0.502, *T*_max_ = 0.715
8995 measured reflections
3214 independent reflections
2928 reflections with *I* > 2σ(*I*)
*R*_int_ = 0.017

#### Refinement

*R*[*F* ^2^ > 2σ(*F* ^2^)] = 0.021
*wR*(*F* ^2^) = 0.061
*S* = 1.05
3214 reflections
200 parameters
1 restraint
H atoms treated by a mixture of independent and constrained refinement
Δρ_max_ = 0.52 e Å^−3^
Δρ_min_ = −0.29 e Å^−3^

### 

Data collection: *APEX2* (Bruker, 2007 ▶); cell refinement: *SAINT* (Bruker, 2007 ▶); data reduction: *SAINT*; program(s) used to solve structure: *SHELXS97* (Sheldrick, 2008 ▶); program(s) used to refine structure: *SHELXL97* (Sheldrick, 2008 ▶); molecular graphics: *X-SEED* (Barbour, 2001 ▶); software used to prepare material for publication: *publCIF* (Westrip, 2008 ▶).

## Supplementary Material

Crystal structure: contains datablocks global, I. DOI: 10.1107/S1600536808022022/hg2424sup1.cif

Structure factors: contains datablocks I. DOI: 10.1107/S1600536808022022/hg2424Isup2.hkl

Additional supplementary materials:  crystallographic information; 3D view; checkCIF report

## Figures and Tables

**Table:** 

Cu1—O1	1.905 (1)
Cu1—N1	1.963 (2)

**Table:** 

O1—Cu1—N1	91.28 (6)
O1—Cu1—N1^i^	88.72 (6)

Symmetry code: (i) 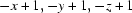.

**Table 2:** Hydrogen-bond geometry (Å, °)

*D*—H⋯*A*	*D*—H	H⋯*A*	*D*⋯*A*	*D*—H⋯*A*
N2—H2N⋯O1^i^	0.88 (1)	2.07 (2)	2.722 (2)	130 (2)

Symmetry code: (i) 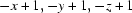.

**Fig. 1.**
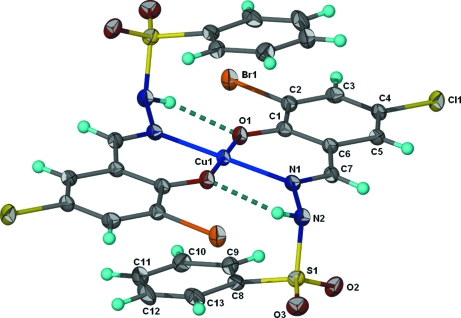
Thermal ellipsoid plot (Barbour, 2001) plot of Cu(C13H9BrClN2O3S)2 at the 70% probability level. Hydrogen atoms are drawn as spheres of arbitrary radius. Hydrogen bonds are denoted by dashed lines. The copper atom lies on a center of inversion, and unlabeled atoms are related to their labeled equivalents by 1 – x, 1 – y, 1 – z.

## supplementary crystallographic information

### Comment

The present study continues with a study on the copper derivative of Schiff-base condensation products from the reaction between substituted salicylaldehydes and benzene sulfonylhydrazine. The two monoanions chelate to the copper atom, which shows square-planar coordination (Ali *et al.*, 2007). The present copper derivative (Scheme I, Fig. 1), which has a bromine substituent in the aromatic system, shows such a geometry; the nature of the substituent in the salicylaldehyde portion does not have an effect on the overall geometry.

### Experimental

3-Bromo-5-chlorobenzaldehyde (0.5 g, 0.3 mmol) and benzenesulfonylhydrazine (0.5 g, 0.3 mmol) were condensed in refluxing ethanol (100 ml) for two hours. The solvent was removed to give the Schiff base, which was collected and dried. The ligand (0.6 g, 2 mmol) and copper acetate (0.2 g, 1 mmol) were heated in ethanol (100 ml) for two hours. The solvent was removed and the product recrystallized from DMSO.

### Refinement

Carbon-bound H-atoms were placed in calculated positions (C—H 0.95 Å) and were included in the refinement in the riding model approximation, with *U*(H) set to 1.2*U*_eq_(C). The amino H atom was refined with a distance restraint of N–H 0.88±0.01 Å; its temperature factor was freely refined.

### Crystal data

**Table:**

[Cu(C_13_H_9_BrClN_2_O_3_S)_2_]	*Z* = 1
*M**_r_* = 840.82	*F*_000_ = 415
Triclinic, *P*1	*D*_x_ = 1.981 Mg m^−^^3^
Hall symbol: -P 1	Mo *K*α radiation λ = 0.71073 Å
*a* = 8.0688 (1) Å	Cell parameters from 5423 reflections
*b* = 8.2755 (1) Å	θ = 2.7–28.4º
*c* = 11.7386 (2) Å	µ = 4.00 mm^−^^1^
α = 95.955 (1)º	*T* = 100 (2) K
β = 90.133 (1)º	Block, purple
γ = 115.159 (1)º	0.20 × 0.09 × 0.09 mm
*V* = 704.70 (2) Å^3^	

### Data collection

**Table:**

Bruker SMART APEX diffractometer	3214 independent reflections
Radiation source: fine-focus sealed tube	2928 reflections with *I* > 2σ(*I*)
Monochromator: graphite	*R*_int_ = 0.017
*T* = 100(2) K	θ_max_ = 27.5º
ω scans	θ_min_ = 1.8º
Absorption correction: Multi-scan(SADABS; Sheldrick, 1996)	*h* = −8→10
*T*_min_ = 0.502, *T*_max_ = 0.715	*k* = −10→10
8995 measured reflections	*l* = −15→15

### Refinement

**Table:**

Refinement on *F*^2^	Secondary atom site location: difference Fourier map
Least-squares matrix: full	Hydrogen site location: inferred from neighbouring sites
*R*[*F*^2^ > 2σ(*F*^2^)] = 0.021	H atoms treated by a mixture of independent and constrained refinement
*wR*(*F*^2^) = 0.061	*w* = 1/[σ^2^(*F*_o_^2^) + (0.0332*P*)^2^ + 0.5714*P*] where *P* = (*F*_o_^2^ + 2*F*_c_^2^)/3
*S* = 1.05	(Δ/σ)_max_ = 0.001
3214 reflections	Δρ_max_ = 0.52 e Å^−^^3^
200 parameters	Δρ_min_ = −0.29 e Å^−^^3^
1 restraint	Extinction correction: none
Primary atom site location: structure-invariant direct methods	

### Fractional atomic coordinates and isotropic or equivalent isotropic displacement parameters (Å^2^)

**Table:**

	*x*	*y*	*z*	*U*_iso_*/*U*_eq_	
Br1	0.46994 (3)	0.68016 (3)	0.125566 (17)	0.01926 (7)	
Cu1	0.5000	0.5000	0.5000	0.01271 (9)	
Cl1	−0.18066 (7)	0.07143 (7)	0.02818 (4)	0.02134 (12)	
S1	0.09184 (7)	0.33794 (7)	0.69365 (4)	0.01590 (11)	
O1	0.4287 (2)	0.53987 (19)	0.35457 (12)	0.0156 (3)	
O2	−0.0713 (2)	0.2946 (2)	0.62483 (13)	0.0201 (3)	
O3	0.0825 (2)	0.2833 (2)	0.80598 (13)	0.0223 (3)	
N1	0.2591 (2)	0.2994 (2)	0.51073 (14)	0.0139 (3)	
N2	0.2075 (2)	0.2348 (2)	0.62019 (14)	0.0149 (3)	
H2N	0.309 (2)	0.267 (4)	0.663 (2)	0.025 (7)*	
C1	0.2899 (3)	0.4324 (3)	0.28525 (17)	0.0142 (4)	
C2	0.2784 (3)	0.4722 (3)	0.17138 (17)	0.0150 (4)	
C3	0.1374 (3)	0.3636 (3)	0.09341 (17)	0.0165 (4)	
H3	0.1350	0.3938	0.0177	0.020*	
C4	−0.0018 (3)	0.2085 (3)	0.12702 (17)	0.0161 (4)	
C5	0.0002 (3)	0.1631 (3)	0.23553 (17)	0.0162 (4)	
H5	−0.0958	0.0571	0.2570	0.019*	
C6	0.1451 (3)	0.2739 (3)	0.31582 (16)	0.0140 (4)	
C7	0.1325 (3)	0.2211 (3)	0.42942 (17)	0.0151 (4)	
H7	0.0238	0.1219	0.4461	0.018*	
C8	0.2345 (3)	0.5685 (3)	0.69679 (18)	0.0168 (4)	
C9	0.2357 (3)	0.6526 (3)	0.60000 (18)	0.0175 (4)	
H9	0.1500	0.5902	0.5372	0.021*	
C10	0.3634 (3)	0.8287 (3)	0.5961 (2)	0.0215 (4)	
H10	0.3658	0.8877	0.5305	0.026*	
C11	0.4881 (3)	0.9189 (3)	0.6887 (2)	0.0254 (5)	
H11	0.5770	1.0388	0.6856	0.030*	
C12	0.4833 (3)	0.8350 (3)	0.7848 (2)	0.0269 (5)	
H12	0.5673	0.8987	0.8481	0.032*	
C13	0.3572 (3)	0.6584 (3)	0.79044 (19)	0.0216 (4)	
H13	0.3547	0.6004	0.8566	0.026*	

### Atomic displacement parameters (Å^2^)

**Table:**

	*U*^11^	*U*^22^	*U*^33^	*U*^12^	*U*^13^	*U*^23^
Br1	0.02148 (12)	0.01787 (11)	0.01395 (11)	0.00366 (9)	−0.00187 (8)	0.00401 (7)
Cu1	0.01245 (17)	0.01403 (16)	0.00878 (16)	0.00304 (13)	−0.00122 (12)	0.00080 (12)
Cl1	0.0154 (2)	0.0276 (3)	0.0122 (2)	0.0018 (2)	−0.00463 (18)	−0.00208 (19)
S1	0.0156 (2)	0.0193 (2)	0.0112 (2)	0.0056 (2)	0.00112 (18)	0.00311 (18)
O1	0.0151 (7)	0.0171 (7)	0.0111 (7)	0.0035 (6)	−0.0023 (5)	0.0018 (5)
O2	0.0148 (7)	0.0249 (8)	0.0179 (7)	0.0054 (6)	0.0001 (6)	0.0047 (6)
O3	0.0253 (8)	0.0291 (8)	0.0123 (7)	0.0107 (7)	0.0031 (6)	0.0054 (6)
N1	0.0167 (9)	0.0141 (8)	0.0095 (8)	0.0052 (7)	0.0006 (6)	0.0018 (6)
N2	0.0160 (9)	0.0166 (8)	0.0106 (8)	0.0051 (7)	−0.0009 (6)	0.0029 (6)
C1	0.0146 (10)	0.0162 (9)	0.0121 (9)	0.0074 (8)	−0.0016 (7)	−0.0007 (7)
C2	0.0161 (10)	0.0148 (9)	0.0134 (9)	0.0061 (8)	0.0005 (8)	0.0012 (7)
C3	0.0181 (10)	0.0214 (10)	0.0111 (9)	0.0096 (9)	−0.0002 (8)	0.0014 (8)
C4	0.0132 (10)	0.0196 (10)	0.0129 (9)	0.0060 (8)	−0.0037 (7)	−0.0043 (7)
C5	0.0145 (10)	0.0171 (9)	0.0149 (9)	0.0055 (8)	0.0002 (8)	−0.0014 (7)
C6	0.0147 (10)	0.0158 (9)	0.0112 (9)	0.0064 (8)	−0.0006 (7)	0.0003 (7)
C7	0.0155 (10)	0.0140 (9)	0.0139 (9)	0.0048 (8)	0.0003 (8)	0.0005 (7)
C8	0.0162 (10)	0.0177 (9)	0.0158 (9)	0.0074 (8)	0.0002 (8)	−0.0015 (8)
C9	0.0160 (10)	0.0184 (10)	0.0165 (10)	0.0062 (8)	−0.0006 (8)	0.0001 (8)
C10	0.0200 (11)	0.0198 (10)	0.0242 (11)	0.0079 (9)	0.0035 (9)	0.0027 (9)
C11	0.0190 (11)	0.0190 (11)	0.0330 (13)	0.0053 (9)	0.0000 (10)	−0.0058 (9)
C12	0.0247 (12)	0.0260 (12)	0.0257 (12)	0.0101 (10)	−0.0090 (9)	−0.0118 (9)
C13	0.0249 (12)	0.0246 (11)	0.0169 (10)	0.0137 (9)	−0.0039 (9)	−0.0047 (8)

### Geometric parameters (Å, °)

**Table:**

Br1—C2	1.890 (2)	C3—H3	0.9500
Cu1—O1	1.905 (1)	C4—C5	1.367 (3)
Cu1—O1^i^	1.905 (1)	C5—C6	1.415 (3)
Cu1—N1^i^	1.963 (2)	C5—H5	0.9500
Cu1—N1	1.963 (2)	C6—C7	1.436 (3)
Cl1—C4	1.744 (2)	C7—H7	0.9500
S1—O3	1.4297 (15)	C8—C9	1.391 (3)
S1—O2	1.4309 (16)	C8—C13	1.393 (3)
S1—N2	1.6945 (19)	C9—C10	1.387 (3)
S1—C8	1.756 (2)	C9—H9	0.9500
O1—C1	1.306 (2)	C10—C11	1.393 (3)
N1—C7	1.296 (3)	C10—H10	0.9500
N1—N2	1.437 (2)	C11—C12	1.376 (4)
N2—H2N	0.88 (1)	C11—H11	0.9500
C1—C6	1.418 (3)	C12—C13	1.390 (3)
C1—C2	1.421 (3)	C12—H12	0.9500
C2—C3	1.377 (3)	C13—H13	0.9500
C3—C4	1.395 (3)		
			
O1—Cu1—O1^i^	180.000 (1)	C5—C4—Cl1	119.90 (16)
O1—Cu1—N1	91.28 (6)	C3—C4—Cl1	119.02 (15)
O1—Cu1—N1^i^	88.72 (6)	C4—C5—C6	120.01 (19)
O1^i^—Cu1—N1^i^	91.28 (6)	C4—C5—H5	120.0
O1^i^—Cu1—N1	88.72 (6)	C6—C5—H5	120.0
N1^i^—Cu1—N1	180.0	C5—C6—C1	120.83 (18)
O3—S1—O2	120.99 (10)	C5—C6—C7	116.47 (18)
O3—S1—N2	104.25 (9)	C1—C6—C7	122.65 (18)
O2—S1—N2	105.94 (9)	N1—C7—C6	124.48 (19)
O3—S1—C8	110.55 (10)	N1—C7—H7	117.8
O2—S1—C8	109.09 (10)	C6—C7—H7	117.8
N2—S1—C8	104.59 (10)	C9—C8—C13	121.3 (2)
C1—O1—Cu1	128.38 (13)	C9—C8—S1	118.74 (16)
C7—N1—N2	114.29 (17)	C13—C8—S1	119.62 (17)
C7—N1—Cu1	126.86 (14)	C10—C9—C8	119.2 (2)
N2—N1—Cu1	118.75 (12)	C10—C9—H9	120.4
N1—N2—S1	112.10 (13)	C8—C9—H9	120.4
N1—N2—H2N	107.8 (18)	C9—C10—C11	119.9 (2)
S1—N2—H2N	103.9 (18)	C9—C10—H10	120.1
O1—C1—C6	124.07 (18)	C11—C10—H10	120.1
O1—C1—C2	119.77 (18)	C12—C11—C10	120.3 (2)
C6—C1—C2	116.16 (18)	C12—C11—H11	119.9
C3—C2—C1	122.80 (19)	C10—C11—H11	119.9
C3—C2—Br1	119.33 (15)	C11—C12—C13	120.9 (2)
C1—C2—Br1	117.84 (15)	C11—C12—H12	119.5
C2—C3—C4	119.11 (18)	C13—C12—H12	119.5
C2—C3—H3	120.4	C12—C13—C8	118.4 (2)
C4—C3—H3	120.4	C12—C13—H13	120.8
C5—C4—C3	121.08 (19)	C8—C13—H13	120.8
			
N1^i^—Cu1—O1—C1	163.78 (17)	C4—C5—C6—C7	177.20 (19)
N1—Cu1—O1—C1	−16.22 (17)	O1—C1—C6—C5	−179.35 (19)
O1—Cu1—N1—C7	11.64 (18)	C2—C1—C6—C5	0.6 (3)
O1^i^—Cu1—N1—C7	−168.36 (18)	O1—C1—C6—C7	3.4 (3)
O1—Cu1—N1—N2	−164.50 (14)	C2—C1—C6—C7	−176.57 (19)
O1^i^—Cu1—N1—N2	15.50 (14)	N2—N1—C7—C6	173.81 (18)
C7—N1—N2—S1	−84.65 (18)	Cu1—N1—C7—C6	−2.5 (3)
Cu1—N1—N2—S1	91.96 (14)	C5—C6—C7—N1	174.74 (19)
O3—S1—N2—N1	−169.59 (13)	C1—C6—C7—N1	−7.9 (3)
O2—S1—N2—N1	61.73 (15)	O3—S1—C8—C9	−169.03 (17)
C8—S1—N2—N1	−53.47 (15)	O2—S1—C8—C9	−33.7 (2)
Cu1—O1—C1—C6	11.6 (3)	N2—S1—C8—C9	79.30 (19)
Cu1—O1—C1—C2	−168.43 (14)	O3—S1—C8—C13	17.7 (2)
O1—C1—C2—C3	179.09 (19)	O2—S1—C8—C13	153.05 (17)
C6—C1—C2—C3	−0.9 (3)	N2—S1—C8—C13	−93.98 (19)
O1—C1—C2—Br1	0.9 (3)	C13—C8—C9—C10	0.9 (3)
C6—C1—C2—Br1	−179.07 (14)	S1—C8—C9—C10	−172.22 (17)
C1—C2—C3—C4	0.7 (3)	C8—C9—C10—C11	−0.1 (3)
Br1—C2—C3—C4	178.82 (15)	C9—C10—C11—C12	−1.1 (3)
C2—C3—C4—C5	−0.2 (3)	C10—C11—C12—C13	1.4 (4)
C2—C3—C4—Cl1	179.83 (16)	C11—C12—C13—C8	−0.5 (4)
C3—C4—C5—C6	−0.1 (3)	C9—C8—C13—C12	−0.6 (3)
Cl1—C4—C5—C6	179.93 (15)	S1—C8—C13—C12	172.46 (18)
C4—C5—C6—C1	−0.2 (3)		

Symmetry codes: (i) −*x*+1, −*y*+1, −*z*+1.

### Hydrogen-bond geometry (Å, °)

**Table:**

*D*—H···*A*	*D*—H	H···*A*	*D*···*A*	*D*—H···*A*
N2—H2N···O1^i^	0.88 (1)	2.07 (2)	2.722 (2)	130 (2)

Symmetry codes: (i) −*x*+1, −*y*+1, −*z*+1.
